# Density drives polyandry and relatedness influences paternal success in the Pacific gooseneck barnacle, *Pollicipes elegans*

**DOI:** 10.1186/1471-2148-14-81

**Published:** 2014-04-16

**Authors:** Louis V Plough, Amy Moran, Peter Marko

**Affiliations:** 1 Horn Point Laboratory, University of Maryland Center for Environmental Science, P.O. Box 775, Cambridge, MD 21601, USA; 2 Department of Biological Sciences, University of Hawaii at Manoa, 2500 Campus Road, Honolulu HI 96822, USA

**Keywords:** Multiple paternity, Barnacles, Genetic benefits, Reproductive skew, Mating strategies

## Abstract

**Background:**

Polyandry is a common mating strategy in animals, increasing female fitness through direct (material) and indirect (genetic) benefits. Most theories about the benefits of polyandry come from studies of terrestrial animals, which have relatively complex mating systems and behaviors; less is known about the potential benefits of polyandry in sessile marine animals, for which potential mates may be scarce and females have less control over pre-copulatory mate choice. Here, we used microsatellite markers to examine multiple paternity in natural aggregations of the Pacific gooseneck barnacle *Pollicipes* elegans, testing the effect of density on paternity and mate relatedness on male reproductive success.

**Results:**

We found that multiple paternity was very common (79% of broods), with up to five fathers contributing to a brood, though power was relatively low to detect more than four fathers. Density had a significant and positive linear effect on the number of fathers siring a brood, though this relationship leveled off at high numbers of fathers, which may reflect a lack of power and/or an upper limit to polyandry in this species. Significant skew in male reproductive contribution in multiply-sired broods was observed and we found a positive and significant relationship between the proportion of offspring sired and the genetic similarity between mates, suggesting that genetic compatibility may influence reproductive success in this species.

**Conclusions:**

To our knowledge, this is the first study to show high levels of multiple paternity in a barnacle, and overall, patterns of paternity in *P. elegans* appear to be driven primarily by mate availability. Evidence of paternity bias for males with higher relatedness suggests some form of post-copulatory sexual selection is taking place, but more work is needed to determine whether it operates during or post-fertilization. Overall, our results suggest that while polyandry in *P. elegans* is driven by mate availability, it may also provide a mechanism for females to ensure fertilization by compatible gametes and increase reproductive success in this sessile species.

## Background

Polyandry, when a female mates with more than one male in a single reproductive period, is a common mating strategy among animals [1-3]. However, explanations of mating frequency based on classical sexual selection theory (e.g. [1,4]) suggest that males, but generally not females, should maximize their reproductive success through numerous matings [5]. Furthermore, multiple matings can have significant fitness costs for females such as disease contraction [6], increased predation risk [7,8], and physical injury [9-13]. Nevertheless, many field and experimental studies now show that females commonly mate multiply to acquire sperm from several males [2,3,14-19].

The solution to the apparent paradox of polyandry is that females acquire direct and indirect fitness benefits through multiple matings. Direct fitness benefits, such as increased parental care [20], protection from predators [21,22], and acquisition of nutrient-rich spermatophores or seminal fluid [8,23], have been described in a number of species, most notably in insects [10,21]. In many other taxa, however, there are no obvious direct benefits of polyandry, suggesting that females may receive indirect (or genetic) fitness benefits from multiple matings [2,19,24-26]. In these species, females may mate multiply to increase the likelihood of fertilization by a high quality mate through sperm competition or sperm selection [14,15,27,28] (*i.e.* the ‘good’ genes hypothesis; [18,19]) or to ensure fertilization with a compatible mate (the ‘compatible’ genes hypothesis; [29-32]). In some cases, sperm with genotypes that differ from a particular female’s eggs are more successful at fertilization or may enhance offspring viability [32-34]. In other cases, greater genetic similarity (overall, or at particular gamete recognition loci) is positively associated with fertilization success [35-37].

While polyandry has been examined extensively in behaviorally complex and mobile terrestrial animals (*e.g.* insects, reptiles, and birds), less considered are the conditions that affect the evolution and frequency of polyandry in sessile marine animals, most of which have resource-free mating systems [38,39], in which the benefits of polyandry are primarily indirect and mating is limited by gametic dispersal distances [40-43]. Polyandry may be beneficial (and therefore common) in sessile species if it increases the likelihood of fertilization by either high quality or genetically compatible sperm when pre-copulatory mate choice is limited [38,41,44]. On the other hand, polyandry may be an unavoidable consequence of reproduction in these species, its frequency a reflection of the density of conspecifics and the inability to reject or avoid multiple fertilizations. Among sessile species, barnacles are most unusual in that they copulate, such that mating is likely limited to only a handful of adjacent individuals in most species (but see [45]). Observations of social polyandry (multiple mating attempts) are common in barnacles (e.g. [46]), but genetic analyses of barnacle mating systems are limited and in some cases have yielded contrasting results with respect to the frequency of polyandry. For example, one study found that the prevalence of multiple paternity was related to density but was generally low, suggesting that siring success was mediated by the distance between mates [47], whereas an unpublished study from another non-stalked species reported polyandry in nearly 80% of broods (D.M. Rand, Cited in [47]). A third study examined paternity in broods from physically isolated individuals of the gooseneck barnacle *Pollicipes polymerus,* finding that some individuals had received sperm through the water column, and thus can potentially reproduce with mates outside the range of an extensible penis [45]. Finally, high relatedness and kin aggregation has been observed in *Semibalanus balanoides*[48] which suggests that genetic identity or compatibility may also be important in determining barnacle settlement and possibly reproductive success. Clearly, the drivers of polyandry in barnacles and other sessile marine animals are complex and more information is needed on the relative roles of ecological and genetic factors influencing the evolution of polyandry in these species.

In this study we used microsatellite markers to examine multiple paternity in a natural population of *Pollicipes elegans*, an hermaphroditic intertidal gooseneck barnacle that is found in high-density aggregations on rocky shores of the tropical and subtropical eastern Pacific. Based on the intriguing findings from previous studies of paternity in barnacles [46,47] and evidence of high relatedness within barnacle aggregations [48] we examined the relationship between conspecific density and multiple paternity, the effect of genetic relatedness on proportional siring success, and the extent of relatedness within *P. elegans* aggregations. Though it is difficult to explicitly show the genetic benefits of polyandry (e.g. [38,39,49]), a relationship between relatedness and siring success indicates that genetic identity is important in determining reproductive success in multiply-sired broods, potentially driving the evolution of polyandry in *P. elegans*.

## Results

### Paternity

Paternity analysis of 416 individual larvae from 14 broods and their respective mothers revealed two or more fathers in 11 of the 14 broods analyzed, with the minimum number of fathers ranging from one to five (mean of 3.36; Table 1). In six out of the 14 broods analyzed, there were multiple solutions for the reconstructed paternal genotypes (range of 3 to 427; most had fewer than 30), which were ranked by likelihood, based on Mendelian segregation. There was no effect of sample size on the number of fathers estimated (Pearson’s correlation, sample size vs. number of fathers: 0.049, P = 0.879). In the three cases of single paternity, offspring inherited at least one allele that was not present in the mother’s genotype, demonstrating that self-fertilization was unlikely.

**Table 1:** Sampling densities, sample sizes and paternity results

**Density**	**Brood name**	**Sample size**	**Paternity (fathers)**
2	C4	41	1
4	C1	18	3
4	C2	22	1
12	C9	37	3
	C10	28	1
	C12	40	3
17	C13	34	5
	C15	28	4
	C16	22	4
22	C18	31	4
	C19	37	5
	C22	27	5
44	C3	21	3
	C6	30	5
	**Average**	**29.71**	**3.36**

**Figure 1:**
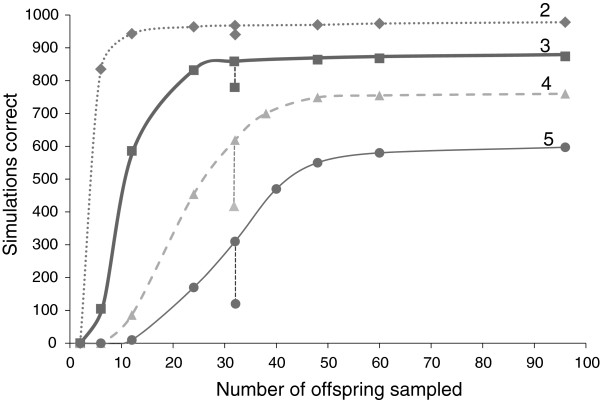
**Power simulation results for detecting multiple paternity with various numbers of fathers (2–5) and offspring sample sizes (6–96) using Gerud2.0.** Simulations correct = the number of iterations (out of 1000) under the given simulation parameters that produced the correct number of fathers. At sample size n = 32, the additional data points represents the power to detect a given number of fathers with skew in paternal contribution (one father sired only 1/5 the offspring compared with the other father (s)).

Our power to detect multiple paternity (two or more fathers) was very high given the markers and sample sizes available in this study. Simulations showed that the power to detect two or three fathers was relatively high (above 0.7) even at low sample sizes of 10 offspring, and increased to 0.97 and 0.84 (for two and three fathers, respectively) when the number of offspring was increased to the mean sample sizes from this experiment (~30; Figure 1). However, power to detect four or five fathers was much lower; at mean sample sizes power was 0.62 and 0.32, respectively. Skew in reproductive contribution (one father contributing only 1/5 the number of offspring) also reduced power substantially in simulations of four and five fathers, but power was not substantially affected for detecting two or three fathers (Figure 1). At the average sample size from this study and the skew scenario evaluated, power to detect four and five fathers with skew was 0.42 and 0.15, respectively.

Among aggregations for which we were able to genotype all of the adults, we found few exogenous alleles in larval broods that could have originated through spermcasting. For the two lowest densities (2 and 4 individuals per 10 cm^2^), densities at which we were able to genotype every individual, we found no external alleles in any larval broods. For one aggregation at a higher density (17 individuals per 10 cm^2^), for which we were able to genotype all but one adult, we found three exogenous alleles in larval broods, suggesting that at least one of these alleles originated from outside the aggregation.

### Effect of density on paternity

**Figure 2:**
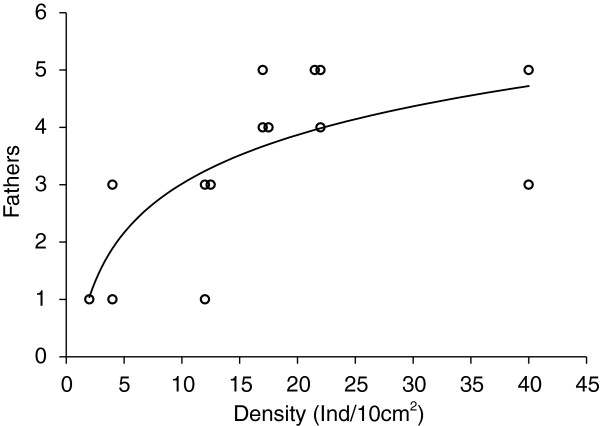
**Scatter plot of density versus paternity.** Curved line indicates the best-fit logarithmic regression line for the data (r^2^ = 0.5122, P = 0.004). Data points are jittered on their x-value to show points that had overlapping x-y coordinates.

Examining paternity in broods sampled from a range of conspecific densities (Table 1; see Methods), we found a positive and significant linear relationship between adult barnacle density and the number of contributing fathers (R^2^ = 0.3521, P = 0.025; Figure 2). This linear relationship appeared to level off with higher density and regression of paternity on log-transformed density produced a better overall fit (R^2^ = 0.513, P =0.0041; Figure 2).

### Reproductive skew and effect of relatedness

**Figure 3:**
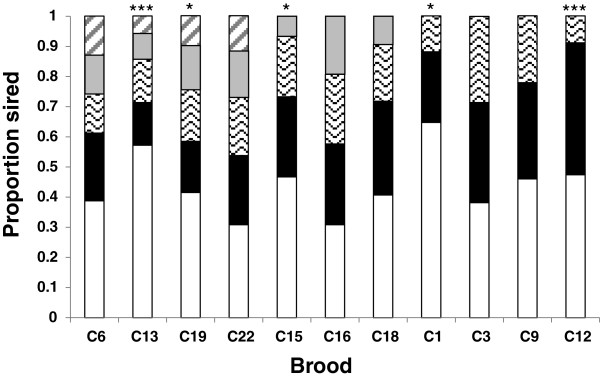
**Proportion of brood sired by putative fathers in multiple paternity broods.** Asterisks indicate significance of chi-square goodness-of-fit tests for equal reproductive contributions of fathers (* P < 0.05, **P < 0.01, ***P < 0.001).

Reproductive contribution of fathers in multiply sired broods was variable, differing significantly from the expectation of equality (evidence of significant reproductive skew) in four of the 11 broods analyzed (Figure 3). In the broods showing significant departures from equal paternal contributions, the patterns of skew were different. For example, in brood c13, most of the offspring (57%) were sired by one individual, while the other four fathers sired somewhat equal (goodness-of-fit Chi-square for the four fathers, P = 0.61), but far smaller numbers of offspring. In brood c12, two of the fathers sired an approximately equal proportion of the offspring (45% each), with the 3rd father siring far less (10%).

**Figure 4:**
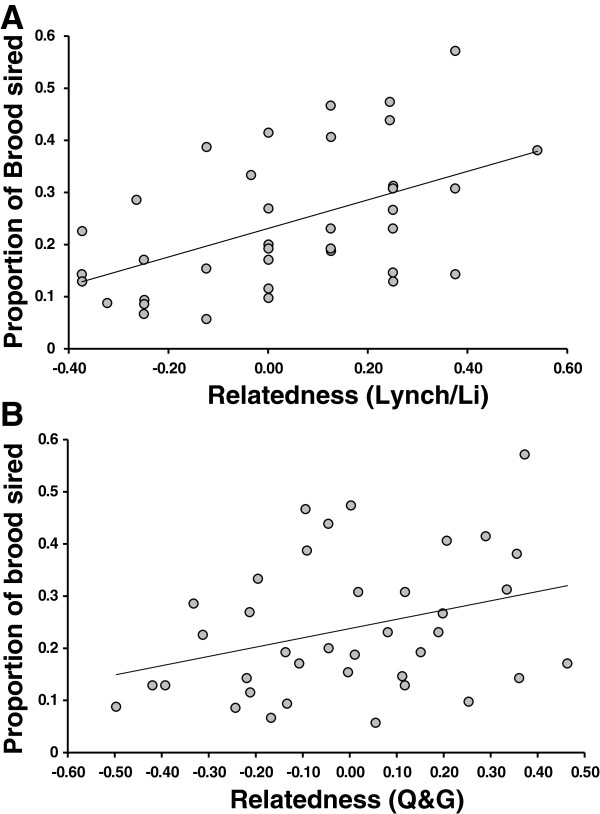
**Scatter plots of relatedness vs. proportion of offspring sired in a brood using two measures of relatedness.** Panel **A**: Relatedness is estimated with the method of Li *et al.* (1993) [78], Panel **B**: Relatedness is estimated with the method of Queller and Goodnight (1989) [50]. Least squares regression lines fit to data for visualization only (see Results for correlations and associated statistical tests).

The permutation-based test of correlation revealed a positive and highly significant association between parental relatedness and the proportion of a given brood sired by putative fathers (Spearman’s Rho = 0.38, P = 0.0084; Figure 4A). This relationship was robust to a number of different relatedness estimators including that of Queller and Goodnight [50] (P = 0.004; Figure 4B). Overall, pairwise relatedness between parents was relatively low (mean = 0.02), though a few parental pairs appeared to show very high relatedness (>0.4; Figure 4).

### Genetic analysis of adult barnacles within aggregations

The eight markers used to examine fine scale population structure and relatedness in the three barnacle aggregations varied in their number of alleles (from four to 12), and showed an overall pattern of high heterozygosity (Additional file 1: Table S1, A1). In aggregation two only, two markers showed significant deviations from Hardy Weinberg equilibrium (Pole 8 and Pole 34; Additional file 1: Table S1). Principal coordinates analysis showed little evidence of allelic differences across the three groups (results not shown). Similarly, overall *F*_ST_ was low (0.007, P = 0.11), with only one significant pairwise *F*_ST_ comparison (aggregation 1 vs 3: *F*_ST_ =0.01, P = 0.003). Relatedness calculations within each group were generally low, (mean = -0.068) with only aggregation one showing a positive average pairwise relatedness (0.008). Relatedness in the first aggregation was significantly greater than expected by chance (P = 0.013) whereas the relatedness estimates for the other two aggregations were not (P > 0.5).

## Discussion

### Multiple paternity in P. elegans

Despite modest sample sizes of offspring and relatively low power to detect four and five fathers, we found a high frequency of polyandry (79% of broods) in *P. elegans*, with up to five fathers per brood. Both the frequency of polyandry and number fathers in multiply-sired brood *P. elegans* are greater than what was found in *Tetraclita rubescens*, an acorn barnacle, which had multiple paternity in 29% of broods and only 4/17 broods with more than two fathers [47], but are similar to unpublished data for another acorn barnacle, *Semibalanus balanoides* (D.M. Rand, unpublished, cited in [47]). These contrasting results suggests that the frequency and extent of polyandry varies substantially among barnacle species, as it appears to vary among other copulating crustaceans such as crabs and lobsters (e.g. [51-55]). Similar variation in the frequency of multiple paternity has been observed across species of birds, in which levels of extra-pair paternity vary from 0% to over 90% [56].

A significant relationship between barnacle density and the number of fathers contributing to broods suggests that the number of potential sperm donors in an aggregation is a significant factor influencing polyandry in *P. elegans*. A similar effect of conspecific density on the frequency of polyandry has been shown in other copulating marine [47,57] and terrestrial [56,58,59] species, but not in a spermcasting, brooding colonial ascidian [43]. Unlike spermcasting invertebrates, most barnacles transfer sperm with an extensible penis that can be several times an individual’s body length, and the ability to successfully fertilize mates is likely determined largely by the length of the penis and the distance to potential mates [60,61], but see [45]. Total extensible penis length is not known for *P. elegans*, but the recent report of relatively short penis length in a related gooseneck barnacle species [45], suggests that mating may also be limited to close neighbors in *P. elegans*.

Most broods possessed microsatellite alleles that matched those found in adults sampled from their mother’s aggregation, indicating that fertilization occurs primarily by physically proximal males. This result suggests that if spermcasting occurs in *P. elegans*, it is only effective over short distances, or within aggregations. While we cannot rule out short-distance spermcasting within aggregations (which may be most effective with mates that are close by, especially in the turbulent, high-energy environments that gooseneck barnacles are found), we found no exogenous paternal alleles in any of the low density aggregations and thus no evidence of long-distance spermcasting. Likewise, most individuals in low-density aggregations lacked lamellae entirely, suggesting that long-distance spermcasting is neither common nor effective in *P. elegans*.

We also observed an apparent upper limit to multiple paternity in *P. elegans*, as indicated by the observation that the linear relationship between the number of fathers siring a brood leveled off at higher densities. Several factors could explain this. First, a lack of power to detect four or more fathers may have produced underestimates of paternity at higher densities resulting in the apparent asymptotic relationship observed between paternity and density. Though power was limited in this study, an upper limit to polyandry may be set by the number of neighbors within reach because penis length is short in *Pollicipes*, and because spermcasting (to the extent that it occurs in this species) may only be effective over short distances. Lastly, females might actively discard sperm or reject copulations after a certain number of insemination attempts, given that copulations can be costly to survival (at least in some species, [9-13]), potentially limiting the number of contributing males at higher densities. In their meta-analysis of multiple paternity in brooding invertebrate species vs. “pregnant” vertebrates [62], Avise et al. noted the far lower levels of multiple paternity than what is theoretically possible given that brood sizes of marine invertebrate species can be as high as 100,000 embryos. An upper limit to the number of fathers contributing to a brood likely represents a balance between the fitness benefits of polyandry and the limitations on multiple mating imposed by logistical or physical factors.

### Polyandry and sexual selection in P. elegans

Evidence of significant skew in paternal contribution and the finding of a significant correlation between genetic similarity and reproductive success suggest that some form of post-copulatory sexual selection may occur in *P. elegans* (though spatial structure in relatedness combined with differential sperm transfer of more proximal neighbors could also produce the observed paternity bias). Paternity bias towards mates with greater genetic similarity could indicate that the specific genetic combination of gametes from the two parents influenced reproductive success, a result that is consistent with a compatible genes model of the benefits polyandry (e.g. [2,3,18]). The finding of a positive relationship between genetic similarity and reproductive success contrasts sharply with much of the current literature on the indirect genetic benefits of polyandry, which typically emphasizes a negative relationship between genetic similarity and reproductive success (e.g. [33,34,63-65]). Mating with genetically dissimilar individuals is thought to be beneficial because increased genetic diversity reduces the risk of inbreeding depression, and ensures a range of genotypes for offspring to contend with environmental uncertainty [2,24]. Other studies have, however, demonstrated fitness benefits of mating between individuals with a high or intermediate level of genetic similarity [18,36,37,66]. Our results show the potential for a similar fitness advantage for fathers that are genetically similar to their mates, supporting the role of compatible genes as a potential benefit of polyandry in this species.

Studies of relatedness in other barnacles may provide clues about why genetically similar mates produce more offspring in multiply-sired broods of *P. elegans*. In the acorn barnacle *Semibalanus balanoides*, barnacle aggregations from a number of rocky outcrops showed higher average relatedness than expected by chance (as high as 0.14) [48], which is likely driven by oceanography (but larval behavior and kin-aggregation may also play a role) and sets the stage for fine-scale spatial variability in relatedness that may be important during reproduction. Though we found that overall genetic similarity was much lower within adult aggregations of *P. elegans* (than compared to *S. balanoides*), our paternity results suggest that during reproduction, there may be a preference for sperm from more genetically similar individuals, even if that similarity is low. Because we could not individually genotype recently-fertilized embryos, it is not clear whether the observed pattern of paternity bias reflects greater fertilization success of compatible gametes or differential post-fertilization viability of compatible embryos. Both scenarios are plausible. Experimental work on the sperm-bindin locus and receptor in various sea urchin species has shown that males with more common or matching bindin genotypes perform better in sperm competition, resulting in greater reproductive success (though this is also mediated by the density of conspecifics and availability of sperm: (e.g. [35,67]). Alternatively, a number of studies show that differential embryo survival influences paternity bias, and its inference [68-70]. Future studies of paternity in barnacles should examine paternity bias and differential reproductive success at different time points—post fertilization (brooding embryos) and during the larval stages—to better understand the role of compatibility during fertilization and during the larval stages.

## Conclusions

In summary, we found that multiple paternity is common in the eastern Pacific gooseneck barnacle *P. elegans* (over 70% of broods and up to five fathers) and that the number of mates contributing to a brood is associated with the density of conspecifics. We also found that higher relatedness between mates conferred an advantage in male reproductive success within multiply-sired broods, suggesting the potential for cryptic female choice of compatible sperm and that polyandry may confer genetic benefits to brooding individuals, though future studies will be needed to test this explicitly. While density clearly affects the availability of potential mates and thus opportunities for multiple paternity, post-copulatory processes (gamete compatibility or cryptic female choice) may be important in ensuring reproductive success in this sessile, polyandrous species.

## Methods

### Sampling and study population

Barnacles were collected from one 20-m-long rocky outcrop, approximately near Punta Gaspareno, Baja California, Mexico (23°10′58.09″N, 110° 8′26.51″W) in October 2011. Aggregations of barnacles at seven different densities—1, 2, 4, 12, 17, 22, and 44 individuals per 10 cm^2^— were sampled haphazardly using a 10 cm^2^ quadrat (see Table 1). We found few individuals brooding embryos in the lowest density groups; only one of eight groups with a density of two individuals per10 cm^2^ and none of the four solitary individuals had embryos. Lower densities (1 or 2 individuals) were sampled more than once in an attempt to find individuals that were brooding embryos for paternity analysis. In total, 119 barnacles were sampled, but of these only 21 individuals exhibited brooding embryos, 14 of which yielded larvae for paternity analyses (Table 1; larval culturing description below). Individual barnacles were scraped off of rocks with 1 mm-thick metal paint scrapers and care was taken minimize damage to the bottom of the peduncle so that adults were brought back alive. Animals from each aggregation were then placed in individual zip-lock bags and kept moist with rinses of fresh seawater every 6–8 hours until they arrived at the laboratory ~24 hours later.

### Larval culturing and tissue sampling

Prior to extraction of the disc-shaped embryo sacs (lamellae), each individual’s peduncle was dipped in a 10% bleach solution, the capitulum was rinsed in 90% ethanol, and then the whole animal was rinsed thoroughly with 35 ppt artificial seawater (ASW, Instant Ocean). Lamellae (both discs) were extracted from the mantle cavity with forceps, rinsed with ASW, and transferred to clean, 100 ul plastic beakers containing 50 ul of fresh ASW treated with 1 mg/l each of streptomycin and penicillin to limit bacterial and fungal growth [71]. Larvae from each brood were reared in separate cultures in the dark at 25°C [71]; each culture was checked daily for hatching. After hatching, swimming larvae were transferred to new beakers and fed with *Rhodomonas salinas* and *Isochrysis galbana* at concentrations of 10,000 cells ml^-1^ each for ~48 h until sufficient numbers of stage-II larvae could be collected (50 or more) and preserved in 70% ethanol. Larvae were fed for 48 h after hatching because larger larvae yielded more DNA, facilitating individual genotyping. Peduncle tissue of mothers with broods that had 50 or more stage-II larvae were sampled and preserved in ethanol.

DNA was extracted from ~25 mg of adult muscle tissue using a modified CTAB protocol (Doyle and Doyle 1987) with two chloroform/isoamyl isolations and two 70% EtOH washes. Precipitated adult DNA was re-suspended with Qiagen EB buffer (10 mM Tris, ph 8.5; Qiagen Valencia CA). DNA from larvae were extracted individually in 200ul 96-well PCR plates using 25 ul of extraction buffer comprised of 0.5% tween, TE buffer (10 mm Tris, 1 mM EDTA), and 2.5 ul 20 mg/ml Proteinase k (Bioline). Larval extractions were incubated at 60°C for 4 hours followed by 30 minutes at 95°C and stored at -20°C. Raw (unprecipitated) larval extractions were used directly in PCR.

### Genotyping and paternity analysis

Brooding individuals (mothers) and offspring were genotyped with at least three of five loci: Pole 1, Pole 8, Pole 25, Pole 29, and Pole 44 [72]. These loci have a high number of alleles (5–23) and gene diversity (average expected heterozgosity = 0.59), show no evidence of null alleles, and exclusion probabilities calculated from the four most commonly genotyped markers (Pole 1, Pole 8, Pole 25, Pole 44) showed high discriminatory power (0.94; genotype data from Gaspareno, Mexico in [72]). PCR was carried out as described in Plough and Marko [72] and fragment analysis was run on the ABI 3100 sequencer at the Arizona State University DNA Lab. Electropherograms were scored by eye using LIZ600 (Applied Biosystems) as an internal size standard on the Peak Scanner software v. 1.0 (Applied Biosystems). Note that because *P. elegans* is hermaphroditic, all individuals, including brooding mothers are potential fathers.

Paternity analysis was performed using the program Gerud 2.0 [73]. The software does not allow for missing data, so only larvae that successfully amplified at all loci were included in the analysis of each brood. In one brood (c22), two larvae (0.48% of all genotyped larvae) were each homozygous at a single microsatellite locus for an allele that differed from the mother’s, violating assumptions of Mendelian segregation. These larvae were removed from the analysis as possible contaminants from another brood but *de-novo* mutation at this locus could also explain the observed segregation pattern (e.g. [74,75]). Multiple genotype array solutions for the fathers were ranked by likelihood using the default test for Mendelian segregation. Though Gerud2.0 has the option of ranking solutions by allele frequencies from a reference or base population, we were not able to use this feature because some of the broods exhibited rare alleles not present in the genotype data from [72]. To determine if offspring were likely sired by fathers in the same physical aggregations, we examined whether the alleles observed in larval broods were also present in the genotypes of adults from the same aggregations in which those broods were collected. We compared larval and adult genotypes from each of two low density aggregations (2 individuals/10 cm^2^ and 4 individuals/10 cm^2^) and eight broods from three high density aggregations (17, 22, and 44 individuals/10 cm^2^; Table 1). All of the adults within the two low-density groups were genotyped, but in the three higher density aggregations we were able to genotype only ~80-95% of the adults because some individuals were damaged during collection and/or transport, compromising the quality of the DNA.

### Power analysis of paternity

To determine the power to detect different levels of multiple paternity given the offspring sample size and the number of contributing fathers, we ran simulations using Gerudsim 2.0. We determined the proportion of simulations out of 1000 that correctly assigned the true number of fathers (from 2–5), given various sample sizes of larvae genotyped (range, two – 96; actual mean sample size across the 14 broods =29.74) and the population allele frequencies of the four most commonly used markers (Pole 1, Pole 8, Pole 25, and Pole 44) from Baja California, Mexico [72]. Simulations were run assuming that the mothers’ genotype was known and that the total offspring number per female (the average number of fertilized eggs) was 5000. In the simulations, five thousand offspring is then split among the true number of fathers for a given simulation scenario (e.g. for the 3 father simulation with equal reproductive contribution, each would be assigned 1666 offspring). Fecundity in *P. elegans* has not been measured systematically, but appears to range from a few thousand to 10’s of thousands of eggs per female based on observations from this study, and estimates for the related barnacle *Pollicipes pollicipes* are similar [76]. Simulations with greater than 5000 total offspring ran extremely slowly in GerudSim2.0, but a few trial runs with 25,000 vs 5,000 total offspring yielded similar power results, so we set the total number of offspring in a brood to 5000. We also determined the power to detect the true number of fathers in a brood when there was skew in paternal contribution: 1/5 the contribution from one father and equal contributions from the others. These simulations were run only at the approximate mean offspring sample size (30). For example, in the case of five fathers, we assigned one father only 200 offspring and the other fathers 1,000.

### Statistical and genetic analyses

Linear regression analysis of the effect of density on paternity was performed in the R statistical software package, v. 2.11.1 [77]. To examine skew in the reproductive contribution of putative fathers, Chi-square goodness-of-fit tests were run in R 2.11.1, with the null hypothesis of equal reproductive contribution. We also examined the association between relatedness of mates (calculated from the reconstructed paternal genotypes and the observed genotypes from the mother of each brood) and proportional paternity success. Relatedness was estimated with the Lynch & Li method [78] in Coancestry[79], because it performs well in a number of situations (e.g. [80]). Given the structure of the data (proportions within each brood sum to 1 and are grouped by female) we used a non-parametric, permutation-based approach to assess the significance of the relationship between relatedness and proportional siring success, because it makes fewer assumptions than analysis of variance or linear mixed-model methods. We implemented a permutation-based test of correlation using Spearman’s Rho (a non-parametric analog to Pearson’s correlation coefficient) that shuffles relatedness values while holding proportion sired static, within each female. The correlation is estimated for the true data, and then calculated after each permutation, and the number of permutations (out of 10,000) in which the permuted value is greater than the actual value is tallied for the one-tailed statistical test. This analysis was performed in R 2.11.1 (see Additional file 2).

Adults from three high density aggregations of barnacles (44, 17, and 22 individuals per 10 cm^2^) were genotyped to assess relatedness among adults and possible fine-scale population structure. The relatedness estimator of Li [78] with weighting by locus [81] was calculated in the program Storm[82]. Storm calculates relatedness within a group or population and uses a permutation procedure to shuffle individuals across populations, creating a distribution of expected relatedness values for each group and overall, against which significance can be assessed. We calculated relatedness in the three aggregations, performing 10,000 permutations to determine if barnacles in these aggregations showed greater relatedness than expected by chance. For these calculations, data from eight microsatellite markers were used (Additional file 1: Table S1). Allele counts, heterozygosities, and tests of Hardy Weinberg Equilibrium were calculated with Arlequin v. 3.5 [83]. We also examined fine scale population structure of the three aggregations using principle coordinates analysis (PCoA) and standard *F-*statistics. PCoA analysis was performed with the Genalex 6.2 software [84], and pairwise and overall *F*_st_ was estimated with Genetix using 10,000 permutations [85].

## Competing interests

The authors declare that they have no competing interests.

## Authors’ contributions

LVP and PM conceived of the study, and LVP carried out all the molecular work and the data analyses. LVP, PM, and AM wrote the manuscript. All authors read and approved the final manuscript.

## Supplementary Material

Additional file 1: Table S1 Population genetic parameters for the three sampled barnacle aggregations (Agg.1-3). Click here for file

Additional file 2 R Code for the correlation function. Click here for file

## Data accessibility

Microsatellite genotype data for all samples have been deposited in Dryad: doi: 10.5061/dryad.ct74m.
