# Supplementary material for: Density drives polyandry and relatedness influences paternal success in the Pacific gooseneck barnacle, Pollicipes elegans
Source: BMC Evol Biol. 2014 Apr 16;14:81. doi: 10.1186/1471-2148-14-81 (PMC4021092; doi:10.1186/1471-2148-14-81)
Supplement: Additional file 1: Table S1 — Population genetic parameters for the three sampled barnacle aggregations (Agg.1-3). [file 1471-2148-14-81-S1.pdf]

Supplementary Table 1

Population genetic parameters for the three sampled barnacle aggregations (Agg.1-3)

		**Agg1**	**Agg2**	**Agg3**
**Pole45**	[a]N	39	15	10
	Alleles	5	4	5
	[b]H_e	0.63703	0.52414	0.63158
	H_o	0.69231	0.46667	0.6
	[d]HWE P-value	0.05795	0.81421	0.88641
**Pole8**	N	41	15	13
	Alleles	7	5	3
	H_e	0.45047	0.65977	0.33538
	H_o	0.39024	0.46667	0.38462
	HWE P-value	0.50906	**0.04706**	1
**Pole29**	N	42	15	14
	A	9	9	8
	H_e	0.86116	0.88276	0.888095
	H_o	0.80952	0.86667	0.78571
	HWE P-value	0.52702	0.64498	0.32938
**Pole25**	N	39	14	11
	Alleles	5	3	2
	H_e	0.48119	0.41534	0.42558
	H_o	0.38462	0.42857	0.54545
	HWE P-value	0.1258	0.62875	0.50428
**Pole34**	N	36	15	14
	Alleles	4	4	4
	H_e	0.61463	0.70115	0.65873
	H_o	0.75	0.66667	0.64286
	HWE p value	0.1258	**0.01712**	1
**Pole1**	N	34	14	14
	Alleles	8	9	11
	H_e	0.76953	0.81217	0.89153
	H_o	0.76471	0.71429	0.78571
	HWE P-value	0.94445	0.40348	0.15841
**Pole44**	N	35	14	10
	Alleles	12	9	8
	H_e	0.83106	0.81217	0.81579
	H_o	0.71429	0.78571	0.8
	HWE P-value	0.05304	0.66405	0.48367
**Pole18**	N	30	14	11
	Alleles	8	4	5
	H_e	0.61808	0.67989	0.68398
	H_o	0.7	0.57143	0.54545
	HWE P-value	0.81486	0.16135	0.30144

[a] number of individuals sampled, [b]H_e and [c]H_o represent expected and observed heterozygosities, respectively, [d]Hardy Weinberg Equilibrium (HWE) test P-value
