# Supplementary material for: Density drives polyandry and relatedness influences paternal success in the Pacific gooseneck barnacle, Pollicipes elegans
Source: BMC Evol Biol. 2014 Apr 16;14:81. doi: 10.1186/1471-2148-14-81 (PMC4021092; doi:10.1186/1471-2148-14-81)
Supplement: Additional file 2 — R Code for the correlation function. [file 1471-2148-14-81-S2.pdf]

Supplementary File 1.

R Code for the correlation function.

```
##example data set to run the model##
ex1 <- data.frame(dam=factor(c(1,1,1,2,2,2,2)),
                  pro.sired=c(.2,.5,.3,.1,.15,.5,.15),
                  rel=c(.2,.4,.1,.05,.04,.1,.5))

##function to calculate correlation from the actual data##
assoc.stat <- function(x) cor(x[,2:3], method="spearman")[1,2]


##permutation function that shuffles relatedness within female (dam) and calculates the correlation ##
permute.once <- function(x) {
  broods <- table(x$dam)
  start.number <- c(0, cumsum(broods[1:(length(broods)-1)]))
  perm <- unlist(sapply(table(x$dam), function(x) sample.int(x,x)))
  perm <- rep(c(0, cumsum(broods)[1:(length(broods)-1)]), times=broods) +
        perm
  x[,3] <- x[perm,3]
  assoc.stat(x)
 }
## block of code to calculate the real correlation and calculate the permuted correlation 10,000 times
## and count the number of times the permuted value is greater than the true value##
 observed.stat <- assoc.stat(ex1)
 null.dist <- double(10000)
 for(i in 1:10000) null.dist[i] <- permute.once(ex1)
 cat("One-tailed P value\n")
 prop.table(table(observed.stat <= null.dist))
```
